# Phloroglucinol-Induced Drug Reaction with Eosinophilia and Systemic Symptoms (DRESS) Syndrome with Subsequent Fulminant Type 1 Diabetes (FT1D): A Rare Case and Literature Review

**DOI:** 10.1155/2024/1018971

**Published:** 2024-09-06

**Authors:** Chengbei Bao, Zequn Tong, Qiuyun Xu, Zhixun Xiao, Bo Cheng, Ting Gong, Chao Ji

**Affiliations:** ^1^ Department of Dermatology The First Affiliated Hospital of Fujian Medical University, 20 Chazhong Road, Fuzhou 350000, Fujian, China; ^2^ Fujian Dermatology and Venereology Research Institute The First Affiliated Hospital Fujian Medical University, Fuzhou, China; ^3^ Key Laboratory of Skin Cancer of Fujian Higher Education Institutions The First Affiliated Hospital Fujian Medical University, Fuzhou 350000, China; ^4^ Central Laboratory The First Affiliated Hospital of Fujian Medical University, 20 Chazhong Road, Fuzhou 350000, Fujian, China

## Abstract

This study reported a woman with drug reaction with eosinophilia and systemic symptom (DRESS) syndrome induced by phloroglucinol who developed fulminant type 1 diabetes as sequelae. The literature review emphasized the necessity of at least seven months of follow-up for better management of DRESS syndrome.

## 1. Introduction

Drug reaction with eosinophilia and systemic symptoms (DRESS) syndrome is a hypersensitivity reaction characterized by fever, facial edema, erupting skin rash, eosinophilia, lymphadenopathy, and internal organ involvement [1]. The most frequently involved organs were the liver (75%), the kidneys (37%), and the lungs (32%) [2, 3]. Hepatic involvement could result in transaminitis, hepatic necrosis, and even failure. Renal involvement could result in elevated creatinine, which may progress to renal failure [3]. Pulmonary involvement could result in interstitial pneumonitis and may progress to acute respiratory distress syndrome and respiratory failure [4]. Gastrointestinal tract, including esophagus, stomach, small intestine, pancreas, and colon, could also be involved in DRESS syndrome, which is less common but also underreported [5]. The acute phase of pancreatic involvement in DRESS can manifest as acute pancreatitis and fulminant type 1 diabetes (FT1D) [6]. It is noted that patients with DRESS syndrome may not only experience internal organ involvement during the acute phase but also develop new-onset diseases due to immune dysregulation after recovery, among which FT1D is rarely reported [7]. Here, we report a case of DRESS syndrome-induced FT1D, review the published cases, and summarize the clinical manifestation thereof.

## 2. Main Text

**Figure 1:**
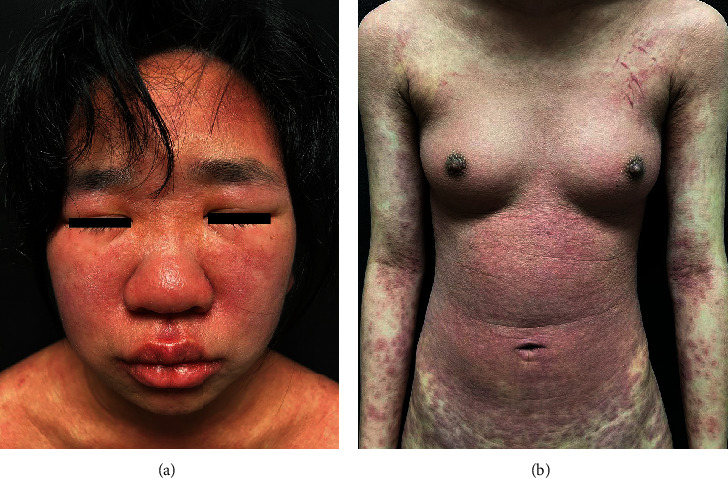
Clinical manifestation of the patient. (a) Facial erythema, edema, and (b) scaly erythematous macules and papules on the trunk and extremities. The total lesions cover over 80% of the body surface area.

**Figure 2:**
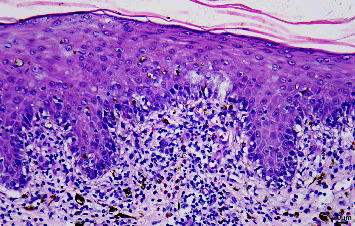
Dermatopathological manifestation of the patient revealed dyskeratosis, interface dermatitis, melanin, and eosinophils in the superficial dermis (H&E ×400).

A 42-year-old woman presented to our hospital complaining of intermittent fever (T max 39.0°C) and malaise. A review of her medical history revealed abdominal pain two weeks ago and a phloroglucinol application for four days. She denied any other medications during this period. The nonpruritic erythematous macules and papules were found on the face and abdomen ten days before, progressing rapidly to the extremities. Physical examination revealed facial erythematous edema, scaly erythematous macules, and papules on the trunk and extremities, covering over 80% of the body surface area (Figures 1(a) and 1(b)). Lymphadenopathy in inguinal regions was palpated. Laboratory tests showed eosinophilia (1.23 × 10^9^/L), liver dysfunction [alanine transaminase 189.7 U/L, aspartate transaminase 136.5 U/L], and elevated inflammatory markers [C-reactive protein 104.8 mg/L, erythrocyte sedimentation rate 34 mm/h]. The following laboratory tests showed EBV DNA of 2.63 *E* + 04 copies/ml, normal antinuclear antibody, blood culture, and hepatitis virus B. Otherwise, the biochemistry, blood glucose, and abdominal ultrasound results were unremarkable. Dermatopathology examination revealed dyskeratosis, interface dermatitis, melanin pigment incontinence, and eosinophils in the superficial dermis, indicating drug-induced manifestation (Figure 2). Based on the RegiSCAR diagnostic criteria, a score of 6 was made, indicating definite drug reaction with eosinophilia and systemic symptoms syndrome (DRESS) syndrome. Methylprednisolone 40 mg intravenously daily was prescribed and gradually tapered after the lesions and liver dysfunction recovered. The patient was discharged with recovery after 20 days of hospitalization.

Twelve days later, the patient experienced muscle weakness and nausea, resulting in an emergency department visit. Laboratory studies showed hyperglycemia (42.6 mmol/L), urine ketone body of 10 mmol/L (3+), urine glucose of 111 mmol/L (4+), metabolic acidosis (pH: 7.34; HCO_3_^−^ 18 mm/L; PCO_2_: 22.5 mmHg), and glycated hemoglobin (HbA_1c_) of 7.6%. Therefore, the diagnosis of diabetic ketoacidosis (DKA) was made, and she was transferred to the Department of Endocrinology. The subsequent test revealed abnormality of autoimmune type 1 diabetes-related antibodies, among which the anti-insulin antibody was 153.80 IU/ml and the antiglutamic acid decarboxylase antibody and the anti-islet cell cytoplasmic antibody were negative. Levels of C-peptide were <0.01 nmol/L in 0′-30″-120′ examination. Pancreatic enzymes were within a normal range. According to the criteria for the diagnosis of FT1D from the Committee of the Japan Diabetes Society [8], the patient was diagnosed with FT1D and insulin replacement therapy was prescribed, which induced rapid remission of symptoms.

## 3. Discussion

DRESS syndrome is characterized by lymphocyte activation, peripheral eosinophilia, reactivation of herpes viruses, and multiorgan involvement. The pathogenesis is hypothesized to be multifactorial, involving a combination of impaired drug detoxification pathways, abnormal immunological reactions, underlying viral reactivation, and genetic susceptibility [4]. Common DRESS syndrome culprits contain anticonvulsant, antimicrobial, antiviral, antidepressant, antihypertensive, biologic, NSAID, and miscellaneous drugs, while no case of phloroglucinol-induced DRESS syndrome has been reported [4]. DRESS syndrome-induced immune system dysregulation may result in several autoimmune sequelae, including autoimmune thyroiditis, reactive arthritis, systemic lupus erythematosus, and diabetes mellitus [9].

FT1D is a subtype of type 1 diabetes mellitus characterized by the rapid onset of ketoacidosis within a few days after developing hyperglycemic symptoms. The pathogenesis of FT1D involves a complex interplay between genetic background, virus infection, and immune dysregulation [10]. Kano et al. [9] reported that the prevalence of FT1D was 3.45% (5/145) according to the survey on sequelae of DRESS syndrome patients. Onuma et al. [11] reported an elevated frequency of FT1D in DRESS syndrome patients compared with the general Japanese population (0.54% vs. 0.010%). Considering the susceptibility of DRESS syndrome-induced FT1D, it is imperative for both physicians and patients to remain aware of regular follow-up care.

We conducted a literature review to summarize the characteristics of DRESS syndrome-induced FT1D. A total of 30 case reports (our case contained) were included (Table 1; see more details in Supplementary Table 1). Onset age ranged from 9 months to 78 years, with the mean age of 52.35 years. The frequent causative drugs included carbamazepine, mexiletine, dapsone, and allopurinol, which were consistent with the common culprits in DRESS syndrome. Endocrine system, digestive system, and cardiovascular system were mostly associated with complications. The involvement of the endocrine system (thyroiditis) and digestive system (pancreatitis) may suggest the development of FT1D in patients with DRESS might share some pathogenesis with the autoimmune polyendocrine syndrome [12]. It is noted that the average interval between the onset of DRESS syndrome and the development of FT1D was 35.51 days (ranging from 0 days to 199 days). Our study highlights the need for long-term follow-up after DRESS syndrome recovery for better management. In light of the findings regarding the time interval, we recommend a 5-week follow-up after DRESS syndrome recovery, followed by ongoing monitoring for at least seven months.

The pathogenesis of DRESS syndrome-induced FT1D was still unclear, although they shared similar pathogenic aspects, including genetic susceptibility, viral infections, and immune dysregulation.

Different HLA haplotype classes were reported in FT1D and DRESS syndrome. Growing evidence has shown the association of specific class II human leukocyte antigen (HLA) haplotypes and FT1D [13, 14]. DRB1∗04 : 05-DQB1∗04 : 01 or DRB1∗09 : 01-DQB1∗03 : 03 were the susceptible haplotypes, while DRB1∗01 : 01-DQB1∗05 : 01, DRB1∗15 : 02-DQB1∗06 : 01, and DRB1∗08 : 03-DQB1∗06 : 01 were the haplotypes resistant to the disease [10, 14]. While the susceptible HLA haplotypes for DRESS syndrome were mainly class I, including HLA-A∗31 : 01, HLA-A∗32 : 01, HLA-B∗13 : 01, HLA-B∗13 : 01, HLA-B∗51 : 01, HLA-B∗58 : 01 [4], in our review, both HLA haplotype classes were involved in DRESS syndrome-induced FT1D. The frequent HLA haplotypes were B62 (5/22), DQB1 (4/22), DR4 (4/22), and DRB1 (3/22), which shows class II haplotype preference. The difference in HLA classes between FT1D and DRESS syndrome patients emphasizes the need for comprehensive HLA haplotype detection in DRESS syndrome patients for better management.

Viral infections were involved in both mechanisms of FT1D and DRESS syndrome [15, 16]. Previous research revealed that HHV-6 infection could strongly induce both proliferation of CD4^+^ and CD8^+^ T cells [17, 18]. Activated T lymphocytes produced large amounts of tumor necrosis factor-*α*, interleukin-2, and interferon-*γ* and considered key mediators of the cytokine release that induces the symptoms found in DRESS syndrome patients [15]. The accelerated innate immune response by viral reactivation may result in the rapid destruction of pancreatic *β*-cells in FT1D with DRESS syndrome [9]. A recent study verified that the reactivation of HHV-6 is associated with the onset of FT1D caused by DRESS [19]. In our review, 86.36% (19/22) of patients reported at least one type of virus reactivation, and HHV-6 was the most implicated trigger, similar to the previous study on DRESS syndrome [4].

## 4. Conclusion

DRESS syndrome could be accompanied by autoimmune sequelae, among which the FT1D was characterized by acute onset and rapid progression of hyperglycemia and DKA. Educating patients to follow up regularly for at least seven months is worthwhile, especially for patients with positive results for viral tests and susceptible HLA haplotypes.

## Figures and Tables

**Table 1:** Clinical characteristics of DRESS-induced FT1D.

Characteristics	Patients (*n* = 30)
Age, median (range) (y)	52.35 (0.75–78)
Sex (male)	43.33% (13/30)
Interval between DRESS and FT1D, average (range) (d)	35.51 (0–199)
HbA_1c_ at onset of FT1D, average (range) (%)	6.70 (5.8–9.2)
Causative drug	*N* = 30 (100%)
Anticonvulsant	*n* = 9
Carbamazepine	6
Lamotrigine, zonisamide, phenytoin	1 for each
Antimicrobial	*n* = 5
Piperacillin/tazobactam, ornidazole, minocycline/cefdynyle^∗^, amoxicillin, and penicillin	1 for each
Antiarrhythmic drug	*n* = 5
Mexiletine	5
Anti-inflammatory drug	*n* = 3
Salazosulphapyridine, diclofenac ibuprofen, acetaminophen	1 for each
Sulfonamides (dapsone)	*n* = 4
Xanthine oxidase inhibitor (allopurinol)	*n* = 3
Other (phloroglucinol)	*n* = 1
Antibody	*N* = 25
GAD	8.69% (2/23)
ICA	0% (0/13)
IAA	25% (1/4)
IA-2	0% (0/2)
Virus reactivation	*N* = 23
HHV-6	75.00% (15/20)
CMV	55.55% (5/9)
EBV	25.00% (1/4)
Cox-B3, Cox-B4, VZV	1 for each
HLA phenotype	*N* = 15
HLA-class I	*n* = 9
B62	5
B44	2
B52	2
B13, B48, BW35, B61, A24, A31	1 for each
HLA-class II	*n* = 13
DQB1	4
DR4	4
DRB1	3
DR2	2
DQA1	2
DR9, DRW12, DRw8	1 for each
Comorbidities	*N* = 26 (86.66%)
Infection	*n* = 2 (7.69%)
Hepatitis C, suppurative tonsillitis	1 for each
Respiratory system involvement	*n* = 3 (11.53%)
ARDS, pneumonia, pulmonary TB	1 for each
Cardiovascular system involvement	*n* = 6 (23.07%)
Atrial fibrillation	2
Arrhythmia, valve replacement, type 2 myocardial infarction, hypovolemic shock	1 for each
Connective tissue diseases	*n* = 3 (11.54%)
Cold agglutinin disease, rheumatic fever, systemic sclerosis-like manifestations	1 for each
Nervous system involvement	*n* = 5 (19.23%)
Subarachnoid hemorrhage, cerebral hemorrhage, convulsion, encephalitis, blepharospasm	1 for each
Endocrine system involvement	*n* = 6 (23.07%)
Thyroiditis	2
Hyperuricemia	2
Type 2 diabetes, IIT	1 for each
Digestive system involvement	*n* = 6 (23.07%)
Pancreatitis	3
Nonalcoholic steatohepatitis, liver dysfunction, liver failure	1 for each
Urinary system involvement	*n* = 3 (11.54%)
Interstitial nephritis	2
Renal failure	1
Skin involvement	*n* = 5 (19.23%)
Livido reticularis, erythema nodosum, vasculitis, photosensitivity, vitiligo	1 for each
Others	*n* = 4 (15.38%)
Alcoholism, schizophrenia, drug addiction, post-therapeutic neuralgia	1 for each

DRESS: drug reaction with eosinophilia and systemic symptoms; FT1D: fulminant type 1 diabetes; GAD: antiglutamic acid decarboxylase antibody; ICA: anti-islet cell cytoplasmic antibody; IAA: anti-insulin antibody; IA2: Insulinoma-associated protein 2 antibody; HHV: human herpesvirus; CMV: cytomegalovirus; EBV: Epstein-Barr virus; VZV: varicella-zoster virus; HLA: human leukocyte antigen; ARDS: acute respiratory distress syndrome; TB: tuberculosis; IIT: iodine-induced thyrotoxicosis. ^∗^Minocycline/cefdynyle could not be identified as culprit drugs in the original case report, but they both belong to the antimicrobial category.

## Data Availability

All datasets generated for this study are included in the article/supplementary material. Further inquiries can be directed to the corresponding author.

## Supplementary Materials

Supplementary Materials Supplementary Table 1 summarizes the published cases of fulminant type 1 diabetes induced by DRESS syndrome.
