# Supplementary material for: Phloroglucinol-Induced Drug Reaction with Eosinophilia and Systemic Symptoms (DRESS) Syndrome with Subsequent Fulminant Type 1 Diabetes (FT1D): A Rare Case and Literature Review
Source: Case Rep Dermatol Med. 2024 Sep 6;2024:1018971. doi: 10.1155/2024/1018971 (PMC11398957; doi:10.1155/2024/1018971)
Supplement: Supplementary Materials — Supplementary Table 1 summarizes the published cases of fulminant type 1 diabetes induced by DRESS syndrome. [file 1018971.f1.pdf]

Supplementary Table 1. Published cases of fulminant type 1 diabetes induced by DRESS syndrome

Authors,	Age, sex	Interval (days)	Drug	HbA_{1c}	Autoantibody	Virus	HLA class I B	HLA class II DR	Co-morbidities	Outcome
Sekine et al. 2001	77, F	14	Carbamazepine	5.9	GAD–; ICA–	HHV6+	N/A	DRB1 DQB1 DQA1	Cold agglutinin disease, Pancreatitis	Survival
Sommers et al. 2002	58, F	11	Allopurinol	6.1	N/A	N/A	N/A	N/A	Pancreatitis	Survival
Seino et al.2004	46, M	2	Mexiletine	6.3	GAD–; ICA–	HHV6+	N/A	DQA1 DQB1	Pancreatitis	Survival
Chiou et al.2006	21, M	60	Diclofenac ibuprofen, Penicillin	N/A	GAD–; ICA–	HHV6+	N/A	N/A	suppurative tonsillitis	Survival
Onuma et al. 2012	70, F	16	Mexiletine	5.8	N/A	HHV6+	B62/B62	DRB1	Arrhythmia	Survival
	46, M	43	Mexiletine	6.7	N/A	HHV6+	B62(5)/B48	DR2/DR4	Type 2 diabetes	Survival
	61, F	20	Mexiletine	7.0	GAD–	HHV6+	B52/B62	DR2/DR4	Rheumatic fever, valve replacement	Survival
	63, M	14	Carbamazepine	6.1	GAD–	HHV6–	N/A	N/A	Post-therapeutic neuralgia	Survival
	19, F	35	Carbamazepine	7.5	GAD–	HHV6+	HLA–B62	DR9	Alcoholism	Survival
	77, F	22	Carbamazepine	6.3	N/A	HHV6+	N/A	DRB1	Schizophrenia	Survival
	31, F	21	Diaminodiphenyl sulfone	N/A	N/A	HHV6+	N/A	N/A	Livedo reticularis	Survival
	60, F	24	Diaminodiphenyl sulfone	6.2	ICA–	CMV+; Cox-B3+	N/A	DR4 DRW12	Erythema nodosum	Survival
	40, M	45	Allopurinol	N/A	ICA–	N/A	BW35	DR4/DRw8	Hyperuricemia	Survival
	69, M	23	Allopurinol	N/A	GAD–	HHV6+; CMV+	N/A	N/A	Atrial fibrillation, hyperuricemia	Survival
	56, M	199	Phenytoin	7.2	GAD–	HHV6–; CMV+	N/A	N/A	Subarachnoid hemorrhage	Survival
	61, M	14	Zonisamide	6.9	GAD–	HHV6+	N/A	N/A	Cerebral	Survival

									hemorrhage, convulsion	
	19, M	89	Salazosulphapyridine	6.6	GAD–	HHV6+	N/A	DRB1	Drug addiction	Survival
	57, F	13	Minocycline or Cefdynyle	5.8	GAD–	HHV6+	B44/B61	DRB1	Hepatitis C	Survival
	72, F	20	Unknown	6.7	GAD–	HHV6+	B13/B62	DRB1	Atrial fibrillation	Survival
Chen et al. 2013	47, F	48	Dapsone	7.2	GAD–	N/A	N/A	N/A	Pulmonary TB, cutaneous vasculitis	Survival
Minegaki et al.2013	71, F	0	Mexiletine	6.0	GAD–; ICA–	HHV6+; CMV+	N/A	N/A	encephalitis, liver dysfunction, acute renal failure, Hashimoto's thyroiditis, and systemic sclerosis-like manifestations.	Survival
Dubois-Laforgue et al. 2013	68, F	120	Amoxicillin Clavulanic acid	7.9	GAD+; IA2–	HHV6–; CMV–; EBV–	N/A	N/A	N/A	Survival
Takeno et al. 2017	78, F	46	Carbamazepine	9.2(steroid–related)	GAD–; ICA–	HHV6–; CMV–; EBV– ; Cox-B4+;	HLA–A24	DRB1 DQB1	blepharospasm	Survival
Marchese et al. 2018	43, M	0	Dapsone	5.9	GAD–; ICA–	N/A	N/A	N/A	Acute interstitial nephritis/ thyroiditis	Survival
Chen et al. 2019	9m, M	6	Piperacillin/tazobactam	N/A	GAD–; ICA–; IAA–;	N/A	N/A	N/A	N/A	Survival
Yuko HIGASHI et al. 2019	47, M	30 (1month)	acetaminophen	N/A	GAD+	N/A	N/A	N/A	Photosensitivity, Vitiligo, Interstitial	survival

								nephritis	
Ping et al. 2019	46, F	14	Ornidazole	6.6	GAD–; ICA–; IAA–;	CMV–	N/A	DRB1 DQB1	IIT	Survival
Pedro Perez et al. 2020	39, F	N/A	Lamotrigine (originated); Amoxicillin (exacerbated)	7.1	GAD–; ICA–	N/A	N/A	N/A	pneumonia, ARDS, nonalcoholic steatohepatitis, hypovolemic shock, liver failure	Survival
Yuki Kobayashi et al.2023	66,M	49 (7 weeks)	Carbamazepine	6.3	ICA-; GAD-; IA2-	VZV+; HHV6-; HHV7-;EBV-;HSV-;CMV-	HLA-A31:01	N/A	type 2 myocardial infarction	Survival
Current case. 2024	62, F	32	phloroglucinol	7.6	GAD–; ICA–; IAA+	CMV–; EBV+	N/A	N/A	–	Survival

Notes: F, female; M, male; HLA, human lymphocyte antigen; N/A, not available or unreported; –, negative; + , positive; GAD, glutamic acid decarboxylase antibodies; ICA, islet cell antibodies; IAA: anti-insulin antibody; IA2, islet antigen 2; HHV-6, human herpes virus 6; CMV, cytomegalovirus; EBV, Epstein-Barr virus; Cox, Coxsackie; ANA, antinuclear antibodies; ARDS: acute respiratory distress syndrome: IIT: iodine-induced thyrotoxicosis
